# Significant response of pulmonary sarcomatoid carcinoma with obstructive atelectasis to treatment with the PD-1 inhibitor camrelizumab combined with transbronchial cryoablation: A case report and literature review

**DOI:** 10.3389/fonc.2022.1013047

**Published:** 2022-10-27

**Authors:** Jiayun Nian, Yong Zhu, Qi Fu, Guowang Yang, Xiaomin Wang

**Affiliations:** Beijing Hospital of Traditional Chinese Medicine, Capital Medical University, Beijing, China

**Keywords:** pulmonary sarcomatoid carcinoma, obstructive atelectasis, camrelizumab, tracheoscopic cryotherapy, case report

## Abstract

Pulmonary sarcomatoid carcinoma (PSC) is a rare subtype of non-small cell lung cancer with high malignancy and poor prognosis. Chemotherapy or radiotherapy do not usually provide satisfactory results in patients with PSC, especially in those with advanced-stage cancer. Targeted therapy and immunotherapy are more precise therapies that may be effective in the treatment of PSC; however, further research is needed. Here, we present a case of stage III PSC with obstructive atelectasis, which is more challenging and hinders treatment. Treatment with the PD-1 inhibitor camrelizumab and transbronchial cryoablation showed significant clinical efficacy. This type of combined treatment has not been reported previously for PSC. Thus, this case may provide a valuable reference for future clinical practice and research.

## Introduction

Pulmonary sarcomatoid carcinoma (PSC) is a unique subtype of lung cancer with high degree of malignancy, poor differentiation, and low incidence that accounts for about 0.4% of non-small cell lung cancer (NSCLC) cases (1). Compared with typical NSCLC, PSC has worse prognosis even at earlier tumor stages (2), with a median overall survival of 10 months and a 15% five-year survival rate (3). The clinical manifestations of PSC are not specific and are generally difficult to identify. Thus, distinguishing between PSC and other types of NSCLC is complicated. Nevertheless, there are differences related to tumor sites and invading structures. The imaging findings of PSC can be central or peripheral (4). When the tumor invades the main tracheal branches, it leads to poor drainage, resulting in obstructive pneumonia or atelectasis, which brings greater challenges to the treatment.

As with other types of NSCLC, early PSC can benefit significantly from surgery. Unfortunately, most patients have middle- or advanced-stage cancer at the time of diagnosis (5). Advanced PSC tends to be resistant to conventional chemotherapy and shows a low response to radiotherapy (6). Immune checkpoint inhibitors such as the inhibitor of programmed death-1 (PD-1)/programmed death-ligand 1 (PD-L1) have significantly improved the clinical outcomes and prognosis of patients with NSCLC. Furthermore, PD-L1 was reported to be highly expressed in PSC tissues (7), showing promising prospects for future studies. Here, we present a patient with PSC and obstructive atelectasis who achieved a significant therapeutic response to camrelizumab (a PD-1 inhibitor) combined with transbronchial cryoablation after 4 months of treatment.

## Case report

**Figure 1:**
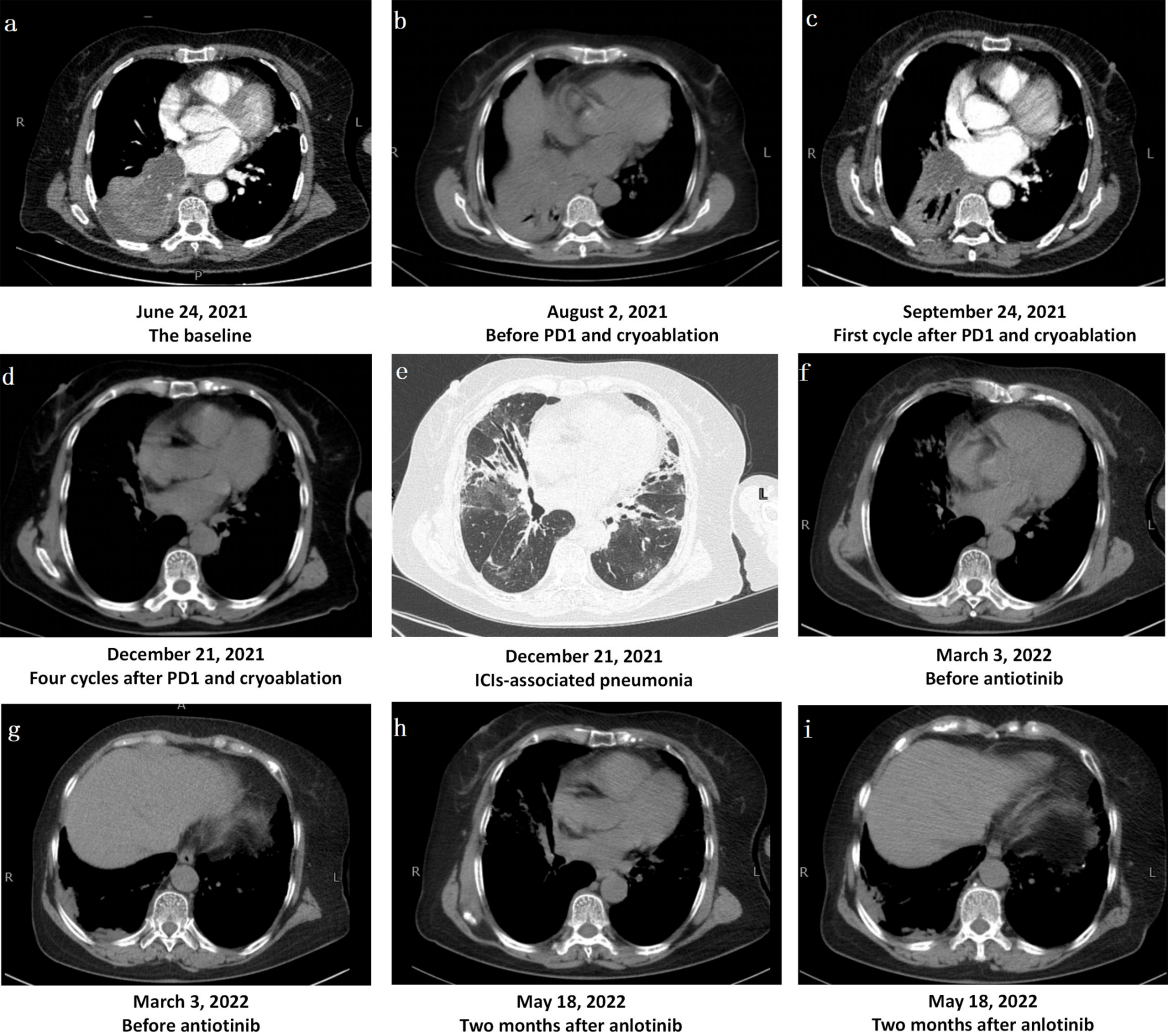
Changes in Computerized Tomography Images. **(A)** The bronchus of the lower lobe of the right lung was truncated from hilus of lung, and the basal segment of the lower lobe of the right lung was clumped with consolidation shadow and obstructive atelectasis. **(B)** Obstructive pneumonia and atelectasis were worse after one-cycle treatment of bevacizumab combined with albumin-bound paclitaxel and carboplatin. **(C, D)** The mass in the lower lobe of the right lung was significantly reduced, obstructive pneumonia and atelectasis were significantly improved after PD1 and chemotherapy. **(E)**After treatment, there were multiple consolidation shadows, ground glass shadows and interstitial changes in chest CT, which was considered as immune checkpoint inhibitor-associated pneumonia. **(F, G)**. After three months of methylprednisolone administration, the tumor near the pleura in the lower lobe of the right lung progressed slightly, and the immune pneumonia was not completely resolved. **(H, I)** After anlotinib treatment, the tumor near the pleura in the lower lobe of the right lung was stable.

**Figure 2:**
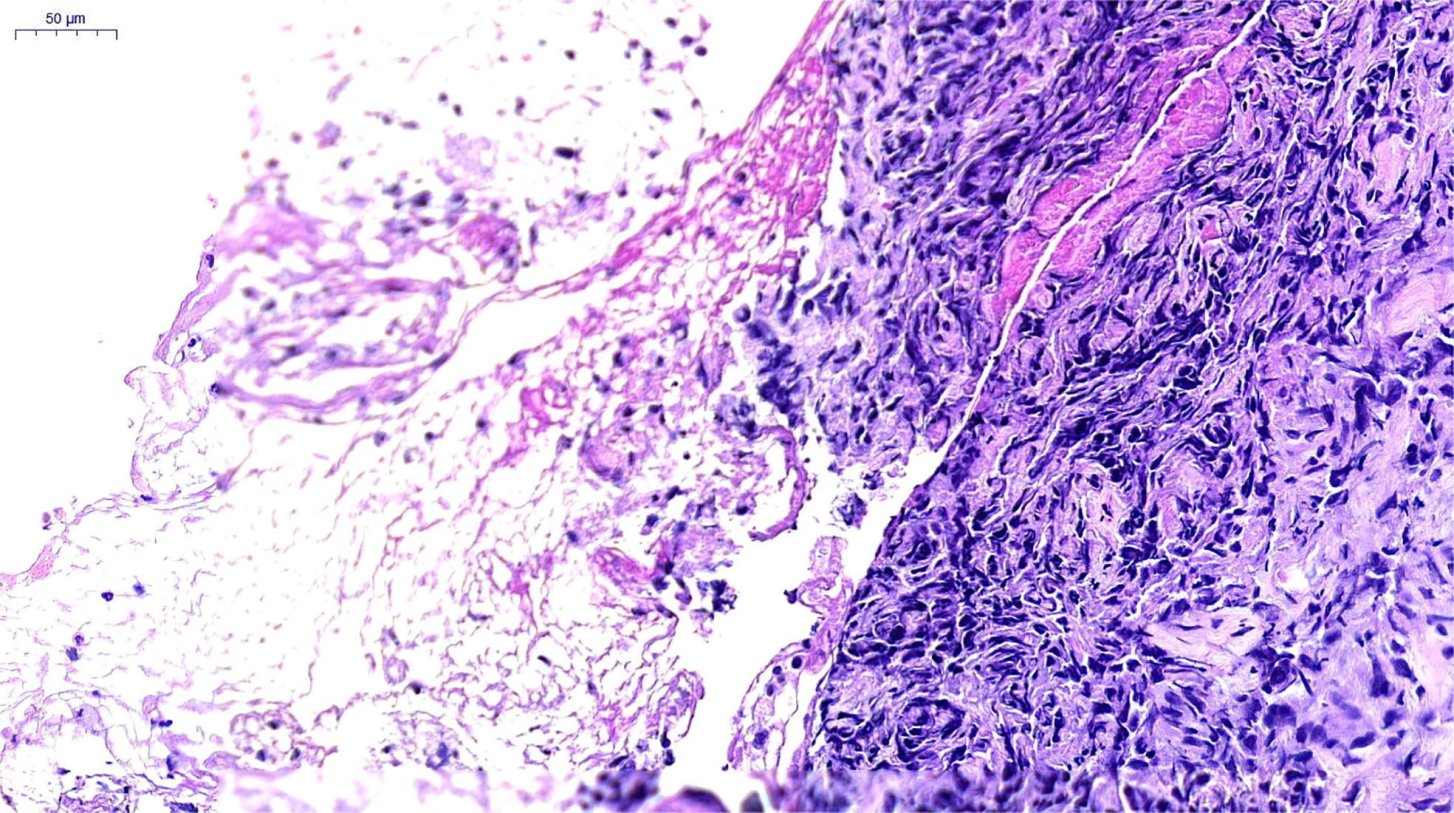
Pathological Findings. Poorly differentiated carcinoma, consistent with pulmonary sarcomatoid carcinoma. H&E×200, hematoxylin and eosin stain.

A 66-year-old female, with a history of Parkinson’s disease and hypertension, suffered from cough, expectoration, hemoptysis, and high fever on June 6, 2021. A chest computerized tomography (CT) revealed a space-occupying lesion in the lower lobe of the right lung. The quinolone antibiotic moxifloxacin was administered as an empirical treatment for community-acquired pneumonia, but the condition did not improve. On June 16, 2021, bronchofibroscopy revealed hyperemic bronchial mucosa in the lower lobe of the right lung, edema, and the formation of new nodules organisms with massive necrosis on the surface. Bronchoalveolar lavage, brushing, and biopsy were performed under tracheoscopy. Suspicious carcinoma cells were found and *Staphylococcus aureus* was cultured in the lavage fluid. However, only a few heterosexual cells and a large number of purulent secretions were found in biopsy tissue. On June 21, she was hospitalized for high fever, cough, expectoration, and asthma. An enhanced CT scanning was performed, indicating malignant lesions in the right lower lobe of the lung with obstructive pneumonia and atelectasis, truncation of the proximal bronchus, invasion of the right pulmonary vein, and multiple ground-glass nodules in both lungs (**Figure 1A**). During her hospitalization, linezolid was given, combined with symptomatic treatments such as resolving phlegm and relieving airway spasm and asthma; however, the clinical symptoms did not improve. A second bronchofibroscopy was performed on July 1, 2021; the middle segment of the right lung was microscopically completely obstructed by neoplasm. The pathological findings of the biopsy tissue suggested poorly differentiated PSC (**Figure 2**). The immunohistochemical results were as follows: Calretinin (weakly +), Syn (−), Ber-EP4 (−), CgA (−), CK5/6 (weakly +), NapsinA (−), Ki-67 (70% +), TTF-1 (−), P40 (−), P63 (−), CD31 (−), CK (AE1/AE3) (+), EMA (weakly +), LCA (−), Vimentin (+). In addition, the tumor stage was T4N0M0. Real-time fluorescence-based quantitative PCR showed no mutations in EGFR, ALK, ROS1, RET, NRAS, and PIK3CA, but mutations in G12A/V/R/C and G13 of KRAS exon 2.

Fever, cough, and wheezing were improved after tracheoscopy, and she began to receive one-cycle treatment of bevacizumab (450 mg/d1) combined with albumin-bound paclitaxel (150 mg/d1, d8, d15) and carboplatin (500 mg/d1) on July 19, 2021. Unfortunately, treatment was suspended on d15 due to worsening symptoms of fever and wheezing, with an ECOG score as high as 3. Re-examination with chest CT showed that obstructive pneumonia and atelectasis were worse than before (**Figure 1B**). On August 10, 2021, she underwent tracheoscopic cryotherapy, expecting to effectively resolve airway obstruction and open a window for further anti-tumor therapy. Tracheoscopic biopsy was performed first, followed by endotracheal carbon dioxide cryotherapy. The probe was sent to the bronchial opening of the middle and lower lobes of the right lung, cryotherapy was respectively performed for two consecutive times, each time lasting 60–120 seconds, and the local temperature reached −60°C at the lowest. After cryotherapy, the middle lobe of the right lung was opened to some extent. Biopsy tissues were taken again for pathological diagnosis and PD-L1 expression levels were determined at the same time. The pathological results supported the diagnosis of PSC and the tumor proportion score (TPS) was as high as 95%.

On August 15, 2021, the patient received the first cycle of camrelizumab (200 mg/d1), albumin-bound paclitaxel (150 mg/d1, d8, d15), and carboplatin (500 mg/d1). During treatment, the patient developed grade III gastrointestinal reactions (WHO classification), presenting with nausea and vomiting, but no recurrence of high fever. After one cycle of treatment, the tumor in the lower lobe of the right lung showed a trend of shrinkage (**Figure 1C**). On the next three cycles, the treatment was adjusted to camrelizumab (200 mg/d1) plus albumin-bound paclitaxel (150 mg/d1, d8, d15) every 21 days, with the last treatment scheduled on November 15, 2021. On December 21, 2021, re-examination with chest CT showed that the mass in the lower lobe of the right lung was significantly reduced, obstructive pneumonia and atelectasis were significantly improved in a short time, and the tumor response evaluation reached partial response (PR) according to the response evaluation criteria in solid tumors (RECIST) version 1.1. However, multiple consolidation shadows, ground glass shadows, and interstitial changes emerged along with tumor shrinking (**Figures 1D, E**). The main clinical manifestations of the patient were shortness of breath, cough, wheezing after activity, but no fever. C-reactive protein, procalcitonin, brain natriuretic peptide, erythrocyte sedimentation rate, G and GM tests, blood culture, bacterial and fungal culture in sputum, sputum acid-fast bacillus, influenza virus RNA, mycoplasma and chlamydia RNA, and other infection-related examinations showed no obvious abnormalities, whereas arterial blood gas analysis suggested hypoxemia with a PaO2 as low as 68 mmHg. After multidisciplinary consultation, immune checkpoint inhibitor-associated pneumonia with grade 2 after PD-1 treatment (Evaluation criteria for common adverse events version 4.0) was considered. Immunotherapy and chemotherapy were discontinued and oral methylprednisolone therapy (56 mg/d, 1 mg/kg/d) was initiated. The dosage of methylprednisolone was gradually reduced and the patient stopped the drug on April 30, 2022. On March 3, 2022, chest CT showed mild tumor progression near the pleura in the lower lobe of the right lung and incomplete remission of immune pneumonia (**Figures 1F, G**). Therefore, a treatment regimen of oral anlotinib (12 mg d1–d14/21 d) was started, and the tumor was stable at the last review in May, 2022 (**Figures 1H, I**).

## Discussion

PSC is a rare and aggressive pathological subtype of NSCLC with poor prognosis and inferior survival outcomes. A study using the National Cancer Data Base showed that the median survival of patients with PSC was short (about half that of patients with other types of NSCLC) and even less than 6 months for stage III-IV tumors (8). Previous studies have shown limited clinical benefit from radiotherapy and chemotherapy in PSC, especially in patients with advanced stages (9, 10). Currently, the treatment of NSCLC has entered the era of immunotherapy and targeted therapy. Nivolumab, as a kind of immune checkpoint inhibitors, was reported to have high response rates and prolonged overall survival in treating PSC,showing a trend toward higher PD-L1 expression in responsive diseases (11). Another two cases were respectively reported the efficacy of tislelizumab (12) and toripalimab (13) in the treatment of PSC. However, there are few studies on PSC and there is no adequate evidence-based conclusion on whether patients with PSC patients benefit from targeted therapy and immunotherapy.

PSC has unique genetic characteristics. The most common genetic changes in PSC include TP53, KRAS mutations, and MET exon-14 skipping, accounting for about 74%, 56%, and 20%, respectively (8, 14–17). This indicates the potential use of novel MET and KRAS inhibitors in the clinical treatment of this disease. KRAS mutation, a factor indicating a poor prognosis (18), has also been reported to be significantly associated with PD-L1 expression in PSC (19). Other types of genetic changes relatively common in NSCLC, such as EGFR, are relatively uncommon in PSC (20). The efficacy of tyrosine kinase inhibitor therapy in patients with PSC and the EGFR mutation is unsatisfactory, suggesting that the EGFR mutation may not be an important driver (21). In terms of immunotherapy, patients with PSC show high tumor mutational burdens and PD-L1 expression (17, 22, 23), which provides a biological basis for the use of immunotherapy against PSC; however, further clinical studies are needed to confirm the results.

The case presented in this article had the following characteristics: first, the patient suffered from a rare late-stage sarcomatoid carcinoma of the lung, which was accompanied by obstructive pneumonia and atelectasis, further increasing the difficulty and challenge of anti-tumor treatment. Second, conventional first-line treatment of advanced NSCLC with platinum-based chemotherapy combined with bevacizumab did not bring clinical benefits. Third, tracheoscopic cryotherapy improved obstructive pneumonia and atelectasis to a certain extent, opening a therapeutic window for antitumor therapy. Fourth, the patient had high PD-L1 expression and received the relatively inexpensive PD-1 drug camrelizumab, which resulted in a significant tumor response and translated into a survival benefit. However, there were limitations in this case. The patient did not undergo a MET mutation test; treatment targeting MET exon-14 skipping could have been beneficial. In addition, the patient did not receive KRAS inhibitors due to drug access issues. PD-1 treatment brought about significant tumor shrinkage; however, it triggered immune pneumonia, which added new challenges to future treatment such as prevention of immunotherapy side effects.

## Conclusion

PSC with obstructive pneumonia and atelectasis is a rare and difficult disease to treat. In this case, both camrelizumab and tracheoscopic cryotherapy were used for treatment. This type of treatment has not been reported in previous studies and achieved good clinical efficacy in this case. Thus, this case may provide a valuable reference for future clinical practice and research.

## Data availability statement

The original contributions presented in the study are included in the article/supplementary material. Further inquiries can be directed to the corresponding authors.

## Ethics statement

Ethical review and approval were not required for the study on human participants in accordance with the local legislation and institutional requirements. The patients/participants provided their written informed consent to participate in this study. Written informed consent was obtained from the individual(s) for the publication of any potentially identifiable images or data included in this article. This report has obtained the written informed consent form the patient’s legal representative (her son) for the publication.

## Author contributions

JN drafted the manuscript. YZ participated in the transbronchial cryoablation and biopsy. QF developed the anti-tumor treatment plan throughout the process. GY and XW both contributed to the conception, design, revision, and final approval of the article. YZ and XW are co-corresponding authors. All authors contributed to the article and approved the submitted version.

## Funding

Beijing Science and Technology Plan: Demonstration and Promotion of TCM Standardized Diagnosis and Treatment of Common Complications of Malignant Tumors (Z191100008319006).

## Acknowledgments

We sincerely thank all those who assisted in this study.

## Conflict of interest

The authors declare that the research was conducted in the absence of any commercial or financial relationships that could be construed as a potential conflict of interest.

## Publisher’s note

All claims expressed in this article are solely those of the authors and do not necessarily represent those of their affiliated organizations, or those of the publisher, the editors and the reviewers. Any product that may be evaluated in this article, or claim that may be made by its manufacturer, is not guaranteed or endorsed by the publisher.
